# 

**Published:** 1857-01

**Authors:** 


PUBLISHED MONTHLY, AT $3 PER ANNUM IN ADVANCE.

### ATLANTA MEDICAL AND SURGICAL JOURNAL.

SMITHSONIAN INSTITUTION

EDITED BY

**JOSEPH P. LOGAN, M. D.,**

Professor of Physiology and General Pathology,

AND

**W. F. WESTMORELAND, M. D.,**

Professor of the Principles and Practice of Surgery.

**Pax et scientia, sed veritas sine timore.**

Vol. II. JANUARY, 1857. No. 5.

ATLANTA, GEORGIA:

PRINTED BY G. P. EDDY & CO.

(Successors to)

C. R. HANLEITER & CO.

1857.

